# Some Statistics in Regard to Tuberculosis

**Published:** 1897-07

**Authors:** 


### ABSTRACTS FROM FOREIGN JOURNALS.

### GERMAN.

UNDER THE DIRECTION OF J. PRESTON HOSKINS,
PRINCETON, N. J.

SOME STATISTICS IN REGARD TO TUBERCULOSIS.—In the first number of the *Archiv fur Thierheilkunde*, for 1897, Dr. Lungwitz, of Grossenhain, Saxony, makes an interesting report in reference to tuberculosis, from which the following is taken:

From June, 1893, until the end of February, 1895, 17,202 full-grown cows (and female calves) were killed in the slaughter-house at Leipzig. Of these 119 were affected with tuberculosis of the udder. In not one of the 119 cases was the udder alone affected. In every case other organs were involved. In 92 cases tuberculosis was general. In the remaining cases it was more or less localized. In only two cases was tuberculosis limited to one other organ besides the udder—in one the bronchial tubes and in the other the mediastinal tubes were involved. From this Dr. Lungwitz draws the conclusion that primary tuberculosis of the udder seldom occurs. The fact that in a period of twenty-one months out of 17,202 cows none were found with tuberculosis of the udder without other organs being involved justifies us in assuming that the occurrence of primary tuberculosis of the udder is very rare. When tuberculosis of the udder is present, tuberculosis of the body is, as a rule, general—that is, distributed over a large number of the organs in several cavities of the body.

As a practical hint for cattle-inspectors he urges that when tuberculosis of the udder is present especial care be exercised in examining the animal for general tuberculosis. Tuberculosis of the uterus is usually connected with abdominal tuberculosis or with general tuberculosis. From August, 1894, until March, 1895, Dr. Lungwitz paid especial attention to the cattle killed at the slaughter-house in Leipzig which were affected with peritoneal or general tuberculosis. In these eight months 267 cases of peritoneal or general tuberculosis were noted; of these 155, or 58 per cent., were affected with tuberculosis of the uterus; 264 of the 267 showed tuberculosis of the peritoneum, and of these 264, 152 (57.9 per cent.) had tuberculosis of the uterus. As his general conclusion from these statistics and his observations, Dr. Lungwitz states that tuberculosis of the uterus usually includes tuberculosis of the oviducts, and is usually accompanied by tuberculosis of the skin of the stomach (Banchfell) without general tuberculosis being present. Tuberculosis of the uterus was also found in calves which had never borne young or been fertilized.

Concerning tuberculosis of the serous membrane in swine, the investigation was in regard to the spread of tuberculosis in the body of swine when tuberculosis of the serous membrane is present. Dr. Lungwitz gathered statistics from July, 1893, until February, 1895. During these twenty months he observed 141 cases of tuberculosis of the serous membrane; 134 times (95 per cent.) general tuberculosis was present. Of the 141 cases the pleuræ were affected 113 times, the peritoneum 15 times, and both pleura and peritoneum 13 times. In all 178,739 swine were killed during this period, and 0.08 per cent. were affected with tuberculosis of the serous membrane.

Dr. Lungwitz sums up his observations in three general conclusions: 1. When tuberculosis of the udder is present in cows the tuberculosis is in almost all cases a general one. 2. That tuberculosis of the uterus in cows is a very common phenomenon, and that it usually involves peritoneal tuberculosis. 3. That tuberculosis in swine is mostly generalized when tuberculosis of the serous membrane is present.

